# Genetic Polymorphisms and Predisposition to Peri-Implantitis: A Systematic Review

**DOI:** 10.3390/ijms262311461

**Published:** 2025-11-26

**Authors:** Filomena Salazar, María Belén Alvarez, Marta Relvas, José Julio Pacheco, Marco Infante da Câmara, José Adriano Costa

**Affiliations:** UNIPRO, Oral Pathology and Rehabilitation Research Unit, University Institute of Health Sciences (IUCS-CESPU), 4585-116 Gandra, Portugal; belenra78@gmail.com (M.B.A.); marta.relvas@iucs.cespu.pt (M.R.); julio.pacheco@iucs.cespu.pt (J.J.P.); marco.camara@iucs.cespu.pt (M.I.d.C.); jose.costa@iucs.cespu.pt (J.A.C.)

**Keywords:** peri-implantitis, genetic factors, genetic predisposition, polymorphisms, dental implants complications

## Abstract

Peri-implantitis is a multifactorial inflammatory disease that can compromise the longevity of dental implants. Several studies have investigated the association between genetic variants. This systematic review aimed to assess the contribution of genetic polymorphisms to the risk of developing peri-implantitis. A systematic search was conducted in the PubMed database up to 2025 following the PICO strategy and PRISMA guidelines. Eligible studies met the following inclusion criteria: articles written in English, addressing genetic associations with peri-implantitis in human subjects, designed as clinical trials or observational (prospective or retrospective) studies, with full-text availability, and published within the last ten years. Exclusion criteria included studies in languages other than English, those not addressing the main research question, publications older than ten years, studies without full text, and secondary research such as meta-analyses or review articles. Selection and data extraction were performed independently by two reviewers. Risk of bias was evaluated qualitatively for control studies. Twenty-three studies met the inclusion criteria. Polymorphisms in genes related to inflammatory cytokines (e.g., *IL-1β* +3954 C/T, *IL-10*, *TNF-α*), bone metabolism (*OPG*, *BMP4*, *FGF10*), and immune regulation (*CD14* rs2569190, *miR-27a-3p*) were frequently reported. *IL-1β* +3954 C/T, *ET-1* and *IL-1β*, *MMP-8* rs11225395 (T allele), *MMP8* (−799 C/T), and *CD14* rs2569190 showed consistent associations with increased peri-implantitis risk, while the results for *TNF-α* −308 G/A were inconsistent across populations. These findings suggest a potential role of genetic predisposition in peri-implantitis development. Identifying genetic biomarkers may help predict susceptibility and personalize management strategies.

## 1. Introduction

The use of osseointegrated dental implants as a method of replacing lost teeth has demonstrated high long-term survival rates [1]. Despite the enormous success achieved, the number of complications, mainly technical and biological, has been steadily increasing [2].

In modern dentistry, biological issues pertaining to osseointegrated implants are a hot topic. Inflammatory diseases linked to bacterial processes are the primary focus of these problems. Two clinical varieties can be distinguished: peri-implant mucositis and peri-implantitis (IP) [2,3].

Only the latter exhibits loss of supporting bone, despite the fact that both disorders share the presence of an inflammatory lesion. The medical disease known as IP affects the tissues surrounding dental implants and is characterized by inflammation of the peri-implant mucosa and a gradual loss of supporting bone [4], which can lead to implant loss or failure [1]. We can consider implant failure when the implant moves, is lost, or there is peri-implant bone loss of more than 1.0 mm in the first year and more than an additional 0.2 mm in the following year [5].

Oral biofilm is the main etiological factor in the development of IP. Just like natural teeth, dental implants provide a hard and non-shedding surface in a fluid environment for the production of biofilms. For instance, poor oral hygiene practices might lead to excessive biofilm production. This may result in inflammation of the tissues around the implant, which may first appear as peri-implant mucositis and then develop into IP [2].

The etiology of IP is complex, while being mostly bacterial. IP can be predisposed by a history of periodontal disease, as harmful bacteria can be found in natural teeth. A higher incidence of IP may also be linked to smoking, diabetes mellitus, cardiovascular disorders, surgical technique, prosthesis design, and excessive cement used in implant rehabilitations [6,7]. Regardless of whether they are intrinsic or modifiable, identifying these factors is extremely important for the prevention and treatment of the disease [5]. It is important to note that not all patients exposed to similar risk factors will develop peri-implantitis, highlighting the importance of genetic predisposition (elucidation of the genetic basis and identification of molecular biomarkers) and differences between populations [8].

Since not all people with the same risk factors develop IP, a genetic predisposition may explain the development of this pathology, although genetic differences are not a known common factor affecting periodontopathogens [4].

Biomarkers in peri-implantology, biochemical and genetic, are a sophisticated tool with the ability to accurately reflect the factors involved in peri-implant pathology, having the ability to compensate for the limitations of other routine diagnostic methods. Genetic biomarkers, which are constant, are suitable for estimating risk and can be measured before the occurrence of the disease; on the other hand, biochemical biomarkers are the biomarkers of choice for monitoring disease onset, intensity, and activity [9].

The most prevalent genetic polymorphisms are single-nucleotide polymorphisms (*SNPs*), which are differences in the DNA sequence that affect over 1% of the population. These changes can be used to determine which genes and proteins are responsible for a certain disease; therefore, studying them enables researchers to examine genetic risk in populations [10,11,12]. The *SNPs* that have been the subject of most research in the context of IP are linked to inflammatory interleukin genes and proteins that are involved in bone metabolism. These genes, when mutated, can induce an anomalous inflammatory response and/or reduced peri-implant osseointegration, resulting in the development of IP. In patients who are at a higher genetic risk for IP, these inflammatory and bone-related molecules may serve as potential biomarkers [11].

As the identification of genetic biomarkers associated with the risk of IP can be extremely important in daily clinical practice, the aim of this systematic review is to evaluate the contribution of genetic characteristics to the development of IP, as well as to identify the possible association between polymorphisms in various genes and predisposition to peri-implant lesions.

## 2. Materials and Methods

A systematic review has been conducted in accordance with the PRISMA 2020 guidelines, in order to identify relevant studies published in the last 10 years. The review was not registered (no protocol registered in PROSPERO, which currently accepts only interventional studies).

### Strategy and Sources

The literature search for this review was carried out in PubMed and the Cochrane database using combinations of the keywords: (periimplantitis [MeSH Terms]) AND (genetics); (peri-implantitis [MeSH Terms]) AND (genetic predisposition); (peri-implantitis [MeSH Terms]) AND (genetic susceptibility); (peri-implantitis [MeSH Terms]) AND (genetic risk factors). Before the literature search, a PICO criteria strategy was implemented (Table 1).

Eligible studies met the following inclusion criteria: (1) articles written in English, (2) articles dealing with genetic associations of IP, (3) human studies, (4) clinical trials, (5) Observational studies, (6) studies with full text available, and (7) articles published within the last 10 years. Exclusion criteria included the following: (1) studies published in languages other than English, (2) studies that did not address the main question, (3) studies published more than 10 years ago, (4) studies with no full text available, (5) meta-analyses, and (6) review articles. Systematic reviews and meta-analyses were excluded from the selection process, as the objective of this work was to synthesize evidence directly from primary studies. The inclusion of secondary analyses could have introduced duplication of data and heterogeneity in interpretation. However, relevant systematic reviews were screened to identify additional primary studies not retrieved through database searches, in accordance with PRISMA recommendations.

The studies that were finally considered eligible addressed the main question: “In individuals who receive dental implants, do genetic alterations increase the susceptibility to developing peri-implantitis compared to individuals without such genetic alterations?”.

The process has entailed several reviewers working autonomously, examining, comparing, and resolving any discrepancies identified.

For each study included, data were systematically extracted regarding the following outcomes: presence or absence of peri-implantitis, genotype and allele frequencies, odds ratios (ORs), and statistical significance of the associations between specific genetic polymorphisms and peri-implant disease susceptibility.

In addition, other study characteristics were collected, including authors, year of publication, country, study design, sample size, population characteristics, and the specific genes or polymorphisms evaluated.

**Figure 1:**
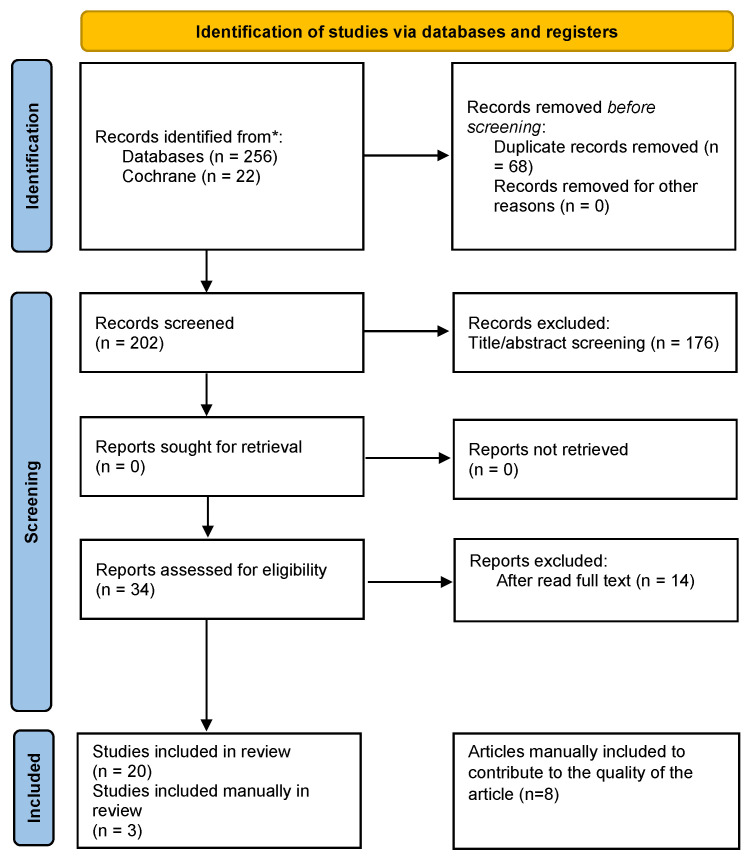
PRISMA 2020 flow diagram for study inclusion. * Automation tools were not used for the study inclusion/exclusion.

The literature search in PubMed and the Cochrane database identified 278 articles. Of these, 68 were excluded as duplicates. The resulting articles were analyzed on the basis of the title and abstract to determine whether they addressed the objective of this review, and 34 were selected, read, and evaluated individually in terms of their purpose. This was complemented by a hand search through selected review articles and reference lists of identified studies for further potentially relevant publications. In total, 20 articles were selected, and 3 were added by manual search, so at the end of the process, 23 were selected (1 population-based study, 4 prospective studies, 3 cross-sectional studies, 15 case–control studies) (Figure 1).

A qualitative synthesis was performed; no quantitative meta-analysis was conducted due to heterogeneity.

## 3. Results

The 23 selected studies included the following: 1 population-based study (investigating whether *CD14*-459 C/T and *TNFα* (−308 A/G) polymorphism are associated with IP); 4 prospective studies (2 of them analyzed the link between *BMP4*, *FGF3*, *FGF10*, and *FGFR1* and polymorphisms in *MMP13*, *TIMP2*, and *TGFB3* genes with IP; 1 investigated the role of chemokine receptor 2 gene polymorphisms in IP susceptibility in a Chinese Han population; and 1 assessed whether there is a solid genetic predisposition that causes the formation of IP in Turkish patients); 3 cross-sectional studies (1 aimed to identify the role of the immune-genetic variation in *IL*-*17A* and related inflammatory cytokine (*IL-23*) in the initiation and progress of IP; 1 assessed the relationships between SNP genotypes, allele-specific risks, the presence or absence of peri-implantitis, and clinical parameter evaluations; and 1 aimed to evaluate the potential of Endothelin-1 (*ET-1*) as a biomarker for diagnosing peri-implant disease); 14 case–control studies (2 of them studied, in Chinese populations, the polymorphisms of the genes *EGF* rs2237051, rs2073617, and rs20773618 (polymorphisms of the *OPG*) and *CD14* with the predisposition to IP; 1 studied, in an Iranian population, the several polymorphisms of *MMPs* with IP and their genetic association; 1 evaluated the expression levels of *IL-4*, *MIP-1α*, and *MMP-9* in healthy peri-implant tissue and IP; 3 conducted studies on healthy and IP patients on the presence and expression levels of various polymorphisms; 1 investigated the relationship between *miR-27a-3p* rs895819 polymorphism and IP susceptibility; 1 analyzed the association between *TNFα* (−308 G/A), *IL-1α* (−889 C/T), and *IL-1β* (+3954 C/T) polymorphisms and the presence/severity of peri-implantitis in Chinese non-smokers; 1 investigated the association between the *MMP8 rs11225395* polymorphism and IP in the Chines Han population; 1 evaluated the existence of a strong genetic predisposition in Turkish patients related to IP risk; 1 assessed whether certain single-nucleotide polymorphisms (*SNPs*) of genes related to inflammation and bone metabolism are associated with the presence of peri-implantitis in the Basque population; 1 analyzed the association of polymorphisms in cytokine genes (*CD14, TNFα, IL6, IL10, IL1ra*) with the risk of peri-implantitis in the Serbian population; 1 investigated polymorphisms in *RANK* (rs3826620), *RANKL* (rs9594738), and *OPG* (rs2073618) and their relationship with mucositis and peri-implantitis in the Brazilian Amazon population; and 1 verified whether individuals with *SNP*s in *IL-1α* and *IL-1β* are more susceptible to develop IP).

The 23 studies included in this review varied in design, population, and genetic markers assessed, encompassing population-based, prospective, cross-sectional, and case–control studies from diverse geographic regions. The risk of bias was generally low to moderate according to the Joanna Briggs Institute (JBI) Critical Appraisal checklist. Most studies presented adequate methodological quality regarding sample selection and outcome measurement, though blinding and sample size justification were often unclear.

### Summary of Evidence Synthesis

No quantitative meta-analysis was performed due to the heterogeneity of genetic markers (same population), study designs (limited sample sizes), uncontrolled confounding factors (history of periodontitis, systemic disease, etc.), and analytical methods (different diagnostic criteria). The synthesis was therefore descriptive. The qualitative evidence indicates that certain polymorphisms—particularly in the *CD14*, *TNFα*, *IL-1*, and *EGF* genes—show consistent associations with increased susceptibility to peri-implantitis, while others (e.g., *MMP13*, *TGFB3*, *RANKL*) demonstrate no clear relationships.

The main results of these studies are presented in Table 2.

A considerable degree of heterogeneity was observed across the studies in terms of population ethnicity, the diagnostic criteria for peri-implantitis, and the genetic loci examined. It is hypothesized that variations in allele frequencies among populations, in conjunction with environmental influences such as smoking or diabetes, may provide a partial explanation for the inconsistencies observed in the reported associations.

In the absence of a quantitative synthesis, formal sensitivity analyses were not conducted. However, the exclusion of studies with a higher risk of bias did not substantially modify the overall interpretation of the evidence, which consistently indicated that genetic factors contribute to peri-implantitis susceptibility.

The following factors were considered for this review: author, title, year of publication, population, type of study, objective of the study, results, and conclusions. Two reviewers independently extracted data using a standardized form. The main characteristics and relevant conclusions of this review are summarized in Table 3. The risk assessment using the Joanna Briggs Institute (JBI) checklist is presented in Table 4.

JBI Critical Appraisal Checklist for Systematic Review Research Syntheses (Control Studies).

## 4. Discussion

Currently, dental implants offer a great treatment alternative for patients with missing teeth by replacing the tooth root with permanent and fixed artificial ones that are placed in the jawbone in line with the natural ones and maintaining the prosthetic crowns [21]. However, despite the high survival and success rate of dental implants, it has long been established that the ability of bone cells to respond to mechanical and biological stimuli is a key factor in successful osseointegration [1,14].

It is suggested that anaerobic bacteria present in plaque could negatively affect the health of peri-implant tissue [2]. Signs and symptoms of IP include redness and swelling, progressive loss of supporting bone, bleeding and/or suppuration on examination, and peri-implant pockets [4,16]. The peri-implant mucosa has a lower regenerative capacity for plaque-related lesions than the gingiva surrounding the tooth, but individuals predisposed to chronic periodontitis are at an elevated risk of developing IP [2].

A successful of an implant depends on several factors, such as the characteristics of the implant (shape, size, connection, surface texture), meticulous planning and organization, the surgery (trauma during surgery, bone quality, specialist experience) [13], risk factors (smoking, oral hygiene, systemic diseases) [6,7,8,12], and prosthetic treatment (overloading, occlusion, and the type of connection between the implant and the prosthesis) [25]. Inadequate control of these factors can cause implant failure.

Prevention and treatment of PI are crucial to ensure the success of the implant. Adopting treatment protocols such as those provided by the EFP (S3 clinical guideline) improves the success rate and reduces complications [26]. Without forgetting the primary prevention of pathologies, identifying all risk factors at an early stage to establish effective prevention strategies. The detection of patients at higher risk enables adequate and individualized planning [27]. To prevent complications in implant placement, one study recommended the use of appropriate treatment techniques and the selection of implants appropriate to each patient’s condition, maintaining proper oral hygiene, and regular check-ups to detect and treat possible complications early [28].

IP, a progressive inflammatory disease that affects hard and soft tissues in the peri-implant space, has been increasingly linked to genetic susceptibility [10,14,15,16]. These genetic markers are crucial in understanding the underlying mechanisms of IP. Groups of genes involved in the regulation of inflammatory responses and bone metabolism have been investigated over time and have been associated with IP, with implications for susceptibility or acceleration of peri-implant tissue destruction [4,9,20].

Cytokines are proteins secreted by leukocytes, and they primarily function as messengers. These cells have the capacity to influence either pro- or anti-inflammatory responses. In the context of peri-implantitis, the equilibrium between pro- and anti-inflammatory cytokines is disrupted, resulting in a pro-inflammatory shift. The most prominent of these are the pro-inflammatory cytokines *IL-6*, *IL-1*, and *TNFα* [29].

Most of the polymorphisms analyzed involve a single-nucleotide polymorphism (*SNP*). In relation to peri-implant disease, genes involved in inflammation and the control of bone metabolism have been examined [10]. The most prevalent gene variations that are subject to examination are genetic polymorphisms in *IL-1α*, *IL-1β*, *TNF-α*, *MMP-8*, and *IL-10* [29]. It is also plausible that certain polymorphisms influence susceptibility to peri-implantitis through transcriptional regulatory mechanisms, such as modifying transcription factor binding or chromatin accessibility at cytokine gene loci. This is similar to observations reported in genome-wide association studies of periodontitis. These regulatory mechanisms are similar to those observed in chronic periodontitis, where genome-wide association studies (GWASs) and *IL-1*-related single-nucleotide polymorphisms (*SNPs*) overlap with DNase I hypersensitivity sites, histone modification marks, and transcription factor binding regions. This influences chromatin accessibility and the transcriptional activation of inflammatory genes. It has been demonstrated that non-coding regulatory variants in *IL-1A*, *IL-1B*, and *IL-1RN* can modulate cytokine expression and immune response profiles by altering transcription factor binding and epigenetic remodeling. Similarly, in peri-implantitis, polymorphisms in cytokine- and matrix-related genes (such as *IL-1β*, *IL-17A*, and *MMP-8*) may exert comparable regulatory effects on gene expression, contributing to dysregulated inflammation and tissue destruction. These findings suggest that periodontitis and peri-implantitis share a molecular framework, in which genetic and epigenetic interactions converge to modulate host susceptibility and disease progression [30].

### 4.1. Polymorphisms

Several polymorphisms in genes involved in inflammatory pathways have been studied to elucidate their role in this disease. Among the potent pro-inflammatory molecules are several cytokines and enzymes involved in the progression of the disease during the host response by triggering inflammation [15,20], in addition to promoting the degradation of extracellular matrix components through matrix metalloproteinases (*MMPs*) where microorganisms in the biofilm stimulate the release of these *MMPs* from the host cells, altering the balance of these enzymes and their tissue inhibitors [7,20].

#### 4.1.1. The Interleukins

Interleukins *(IL*) are a class of cytokines that play critical roles in the peri-implant inflammatory response. The *IL-1β* protein has been shown to regulate the degradation of the extracellular matrix during the process of inflammation. Furthermore, it has been demonstrated that this process induces the production of prostaglandin E2, which in turn affects the degradation of hard tissue [15]. Other ILs, including *IL-6*, *IL-10*, *IL-17*, and *IL33*, have been localized in the peri-implant crevicular fluid. IL-2 and IL-6 are pro-inflammatory cytokines that can stimulate osteoclast activity and bone resorption [2]. Interleukin-10 (*IL-10*), *IL-22*, and other anti-inflammatory cytokines seem to shield supporting tissues from destruction [15,21].

#### 4.1.2. The RANKL

*RANKL*, also known as osteoprotegerin ligand (*OPG*) or *TNF*-related activation-induced cytokine, belongs to the *TNF* superfamily of cytokines. The *RANK-RANKL-OPG* signaling system controls osteoclastogenesis, osteoclast activation, and bone resorption. *RANKL*, an important molecule for bone metabolism, is mainly produced by osteoblasts and fibroblasts. When *RANKL* binds to its transmembrane receptor kappa-B (*RANK*), it increases the resorptive capacity of osteoclasts [4].

#### 4.1.3. The Matrix Metalloproteinases

Matrix metalloproteinases *(MMPs*), biological mediators, are a family of 23 enzymes of similar structure but different genetic origin. They are divided into different groups and are responsible for the degradation of organic components of the extracellular matrix, in both healthy homeostasis (in other words, continuous tissue remodeling) and disease. The activity of these *MMPs* is regulated by tissue inhibitors, called *TIMPs*, which possess an N-terminal domain with the ability to inhibit these *MMPs*. The teaming between *MMPs* and *TIMPs* establishes the level of extracellular matrix breakdown, including hormones, oncogenic substances, anti-inflammatory and pro-inflammatory cytokines, and developmental factors. *MMPs* are present in the peri-implant sulcular fluid and may contribute to bone loss, with several polymorphisms investigated [16]. This potential relationship may help in identifying IP in a timely manner, which may lead to more efficient patient treatment and ultimately improve implant longevity [31].

#### 4.1.4. The Tumor Necrosis Factor

Tumor necrosis factor (*TNF*) is a cytokine that is linked to systemic inflammation. Tumor necrosis factor alpha (*TNF-α*) is a pro-inflammatory mediator that regulates bone resorption and facilitates monocyte–macrophage cell differentiation into osteoclasts, thus promoting osteoclast maturation and enhancing their bone resorption capacities. It is considered a potential predictive marker for disease development [16].

#### 4.1.5. The Bone Morphogenetic Proteins

Bone morphogenetic proteins (*BMPs*) are a group of growth factors that promote osteogenesis and are part of the tumor growth factor-beta (*TGF-β*) superfamily with the ability to induce cartilage and bone formation. The *BMP-4* genetic polymorphism has been linked to marginal bone loss around implants before loading [14].

#### 4.1.6. Other Genes

Other genes regulating inflammatory responses or bone metabolism have been studied in relation to dental implant loss or IP. Differentiation cluster 14 (*CD14*) is involved in the immune response by co-receiving the Toll-like receptor for the detection of bacterial lipopolysaccharide. It is suggested that patients with IP have some genetic variants that induce immunophenotypes characterized by an excessive host response affecting the intensity of inflammatory osteoclastogenesis. Genetic polymorphisms involve different variants of nucleic acid composition and are considered physiological without causing changes in protein function, unlike genetic mutations [9].

### 4.2. Association of Genetic Polymorphisms with Peri-Implantitis

The overall certainty of the evidence was considered low to moderate. While the majority of the included studies exhibited acceptable methodological quality as per the JBI checklist, the heterogeneity in study design, sample size, and analytical approaches constrained the robustness of the conclusions. The absence of standardized diagnostic criteria for peri-implantitis, coupled with the heterogeneity in genetic markers, further diminished the comparability of the findings. Nevertheless, consistent associations were observed for certain polymorphisms—particularly *CD14* rs2569190, *IL-1β* +3954 C/T, and *TNF-α* −308 G/A—across independent populations, supporting a moderate level of confidence in these findings as potential biomarkers for peri-implantitis susceptibility.

This review synthesizes findings from key studies that explore genetic polymorphisms as risk factors for IP in diverse populations. The variation in study outcomes may be attributed to genetic diversity, sample size variation, and environmental or behavioral confounders such as smoking status and oral hygiene practices. Populations from Asia, Europe, and Latin America exhibit divergent allele frequencies for the same *IL-1* polymorphisms, a variation that may partly account for the inconsistent findings. These discrepancies underscore the multifactorial nature of peri-implantitis susceptibility, emphasizing that a single polymorphism cannot account for the observed variations. It is hypothesized that host–microbe interactions, ethnicity, and lifestyle factors likely modulate gene expression and disease severity. Such heterogeneity is consistent with findings in periodontitis, where *IL-1α* and *IL-1β* polymorphisms show population-specific associations influenced by ethnic background and environmental exposure.

A study by Chang et al. (2021) [11] focused on the epidermal growth factor (*EGF*) gene polymorphisms (rs2237051 and rs4444903) with IP in a Chinese Han population. The rs2237051 GG genotype and G allele were identified as protective factors, showing lower levels of peri-implant inflammatory markers and periodontal indexes (gingival index, plaque index, calculus index). However, no significant association was found for the rs4444903 polymorphism, emphasizing the complexity of genetic influences on IP [11]. The authors concluded that the *EGF* rs2237051 polymorphism may play a protective role in the development of IP, whereas Zhou et al. (2016) [4] evaluated the *OPG* gene polymorphisms rs2073617 and rs2073618 in a Chinese Han population. The rs2073618 CC genotype and C allele were associated with a significantly higher risk of IP (OR = 2.18 for CC genotype). The rs2073617 polymorphism did not show a significant correlation with disease susceptibility. The authors concluded that the rs2073618 polymorphism in the *OPG* gene contributes to increased IP risk, emphasizing genetic factors that influence peri-implant bone stability [4].

Ribeiro et al. (2017) [1] conducted a study in the Brazilian population with 90 partially edentulous patients and 245 implants of the same brand in order to assess the association of *IL-10* (−1082 A/G) and *RANKL* (−438 A/G) polymorphisms with implant failure (mobility, recurrent peri-implant infection with suppuration, probing depth ≥ 5 mm or bleeding on probing, continuous radiolucency, subjective complaints). The results demonstrated that the mutant allele (*G*) in *RANKL* was observed in 52.3% of cases, and the mutant allele (*A*) in *IL-10* in 37.8%. Nevertheless, they found no significant association between the genotype or allele frequencies of these *SNPs* and implant failure [1].

Silva et al. (2020) [13] conducted a study of 114 patients with dental implants, classified as healthy, mucositis, and peri-implantitis, analyzing polymorphisms in the *RANK*, *RANKL*, and *OPG* genes using real-time PCR. The findings indicated that factors such as advanced age, thin peri-implant phenotype, biofilm, smoking, alcoholism, and diabetes exhibited a significant association with mucositis and peri-implantitis. However, no statistically significant relationship was found between the genetic polymorphisms studied and peri-implant diseases. The authors conclude that, in this population, clinical and environmental factors have a greater impact than genetic polymorphisms on the onset of peri-implantitis [13].

The tumor necrosis factor-alpha (*TNF-α*) gene polymorphisms have been a subject of mixed results. Rakic et al. (2015) [9] explored the *CD14*-159 C/T and *TNF-α*-308 A/G polymorphisms as potential biomarkers in IP among Southeastern European Caucasians. The *CD14*-159 CC genotype was strongly associated with IP, showing a fivefold increased risk and elevated inflammatory markers such as *RANKL/OPG* ratios. *TNF-α*-308 AG genotype demonstrated a fivefold increased risk of IP. CD14 and TNF-α polymorphisms are significant genetic markers for IP and suggest a hyperactive inflammatory response. This discrepancy may be due to differences in study design, sample size, or environmental factors influencing gene expression, highlighting the need for standardized research protocols [9].

In 2017, Petkovic-Curcin et al. conducted a study involving 98 Serbian patients, in which they evaluated polymorphisms in cytokine genes, including *CD14*, *TNFα*, *IL6*, *IL10*, and *IL1ra*. The results indicated that smoking and the presence of the *TNFα*-308 GA/AA genotype significantly increased the risk of peri-implantitis, while the *CD14*-159 CT/TT polymorphism acted as a protective factor. Correlations with clinical parameters, including probing depth and bleeding on probing, were also identified. The authors concluded that specific polymorphisms, in conjunction with environmental factors such as smoking and a history of periodontitis, may influence the susceptibility to peri-implantitis in this population [8].

In 2020, He K. et al. conducted a case–control study with 318 non-smoking Chinese subjects (144 cases with peri-implantitis, 174 controls with healthy implants) to evaluate the association of genetic polymorphisms with the presence and severity of peri-implantitis. The genetic polymorphisms in question included *TNF-α* (−308 G/A), *IL-1A* (−889 C/T), and *IL-1B* (+3954 C/T). The clinical parameters (probing depth, bleeding on probing, gingival index, plaque index, clinical attachment level) were measured, and the genotyping of the indicated single-nucleotide polymorphisms (*SNPs*) was performed. The results demonstrated that genotypes with the *T* allele in *IL-1A*-889C/T and *IL-1B*+3954C/T were significantly associated with an elevated risk of peri-implantitis and with more unfavorable clinical values. Conversely, the *TNF-α*-308G/A polymorphism exhibited no substantial association. The conclusion drawn from this analysis indicates that *IL-1A* and *IL-1B* single-nucleotide polymorphisms (*SNPs*) could function as genetic markers of predisposition to peri-implantitis in the present population [6].

Studies by Cardoso et al. (2022) and Martins et al. (2022) examined the potential involvement of *IL-1* and cytokines in IP susceptibility [15,21]. Cardoso et al. noted a potential link between *IL-1A* and *IL-1B* polymorphisms and IP, though statistical significance was not achieved [15]. Martins et al., in their analysis of *AhR, IL-6*, and *IL-22* gene expression in peri-implant tissues, observed significantly higher expression of *IL-6* and *AhR* in peri-implant tissues, indicating a heightened inflammatory response. These studies suggest that inflammatory cytokine signaling may be crucial in the pathogenesis of IP [21].

Further research by Turkmen et al. (2022) and Li et al. (2024) focused on other genetic markers like *FcγRIIa*, *FcγRIIIA*, and *CD14* [3,10]. Turkmen and Firatli identified a significant correlation between *FPR1* gene polymorphism and IP [3], while Li et al. linked the *rs2569190 GG* genotype of *CD14* with an increased IP risk in a Chinese Han population [10]. These studies reinforce the idea that immune system-related genes may influence IP susceptibility, particularly through their involvement in inflammatory processes. Similarly, Giro et al. found elevated *IL-4* expression in IP tissues, indicating its role in perpetuating inflammation [2].

*MMPs* play a crucial role in tissue remodeling and inflammation. Saremi et al. (2024) [20] further explored the genetic basis of IP. Saremi et al. found that *MMP-3* and *MMP-7* polymorphisms significantly influenced susceptibility to IP, with the MMP-3 6A allele reducing risk [20]. The *MMP-8* rs11225395 polymorphism, particularly for individuals with the TC/TT genotype, was significantly associated with IP [22]. In addition to the above, *MMP8* with −799C/T polymorphism, and the *T* allele is strongly linked to IP, indicating its potential as a genetic marker for disease susceptibility [23].

Similarly, Talib et al. 2024 found that the *IL-17A* gene polymorphism was associated with increased IP risk, with higher *IL-23* levels contributing to inflammatory processes in peri-implant tissues [17].

On the other hand, a prospective study carried out by Gonçalves Junior et al. (2016) evaluated the possible association between polymorphisms in the *MMP13*, *TIMP2*, and *TGFB3* genes and IP, not finding a significant association between the polymorphisms of the genes studied and the development of IP [7].

Qi et al. (2021) examined the role of *BRINP3* and *CXCR2* gene polymorphisms and observed that the *CT* genotype of *CXCR2* rs2230054 and the *AG* genotype of rs1126580 were more frequent in IP patients, suggesting a role for *CXCR2* in the development of the disease [19].

The study conducted by Saremi et al. (2021) [16] identified a significant association between IP and specific gene polymorphisms. Allele and genotype frequencies of *IL-10* −819C>T, *IL-10* −592C>A, and *IL-1ß* +3954C>T were significantly different in IP patients compared to healthy controls, suggesting these *SNPs* may contribute to IP susceptibility. However, no significant association was found for *TNF-α*-857G>A and *TNF-α*-308G>A polymorphisms. These findings highlight the potential role of specific cytokine gene polymorphisms in the pathogenesis of IP [16].

In 2024, Lafuente-Ibáñez-de-Mendoza et al. conducted a case–control study involving 161 participants (80 with peri-implantitis and 81 controls) from the Basque Country. The aim was to evaluate the association between single-nucleotide polymorphisms (*SNPs*) in genes involved in inflammation and bone metabolism (*BMP-4*, *BRINP3*, *CD14*, *FGF-3*, *FGF-10*, *GBP-1*, *IL-1α*, *IL-1β*, *IL-10*, *LTF*, *OPG*, and *RANKL*) and peri-implantitis. The disease was defined using clinical and radiographic criteria, data on smoking and diabetes were collected, and genotypes were determined using standard blinded methods. *GBP1* rs7911 and *BRINP3* rs1935881 SNPs were found to be significantly more prevalent in patients with peri-implantitis. Additionally, the *OPG* rs2073617 SNP was significant among smokers, and the *BMP-4* rs17563 and *FGF-3* rs1893047 SNPs were significant among patients with type 2 diabetes. These findings suggest that genetic variants may predispose individuals to peri-implantitis by altering the immune response or osseointegration, particularly when combined with risk factors such as smoking or diabetes [12].

A recent study investigated the relationship between the *miR-27a-3p* rs895819 polymorphism and IP susceptibility, demonstrating that the presence of the *GG* genotype (adjusted OR = 2.157, 95% CI = 1.045–4.455) and *G* allele (*p* = 0.003, OR = 1.501, 95% CI = 1.144–1.968) is significantly associated with an increased risk of developing the condition. Additionally, IP patients exhibited decreased *miR-27a-3p* levels and elevated concentrations of *RUNX1*, C-reactive protein (CRP), interleukin-6 (*IL-6*), and white blood cell (WBC) count. These findings suggest that rs895819 may serve as a relevant genetic marker for IP predisposition, influencing the regulation of inflammatory factors and immune response [18]. Further research is warranted on the presence of *ET-1* and its role in the disease. Reporting indicates that the significant increase in its expression in IP mucositis suggests its potential to enable earlier and more accurate assessments of IP inflammation when combined with conventional examination methods [24].

### 4.3. Integrated Scope Analysis

Despite the fact that a number of recent systematic reviews have addressed the genetic association between different polymorphisms and peri-implantitis, a quantitative synthesis (meta-analysis) could not be performed in the present work. The included studies exhibited substantial heterogeneity in study design, diagnostic criteria, and genetic markers evaluated, as well as differences in population ethnicity, allele frequencies, and environmental exposures such as smoking and diabetes. Furthermore, the majority of studies reported incomplete or incomparable data, particularly concerning genotype distribution or odds ratios (OR) with confidence intervals. This precluded consistent statistical aggregation.

The subgroup of studies focusing on cytokine polymorphisms (*IL-1A*, *IL-1B*, *IL-10*, *IL-17A*) demonstrated variable associations with peri-implantitis risk. While *IL-1β* +3954 C/T was repeatedly linked to higher susceptibility, results for *IL-10* and *TNF-α* were inconsistent across populations. Furthermore, the diagnostic criteria for peri-implantitis exhibited significant variability, ranging from radiographic bone loss exceeding 1 mm to pocket depth measuring at least 5 mm. It is noteworthy that adjustments for confounders, such as smoking and a history of periodontitis, were frequently absent. These discrepancies have the effect of limiting the comparability required for pooled estimation.

The evaluation of genetic variations (*OPG* rs2073618, *RANKL* rs9594738, *BMP4* rs2761884) implicated in bone metabolism has been the subject of study in a limited number of small, ethnically diverse cohorts, including Brazilian, Chinese, and Basque samples. The allele frequencies and outcomes (implant failure vs. peri-implantitis) across these groups have been shown to vary significantly. A number of studies reported no statistically significant associations, with methodological designs ranging from cross-sectional to case–control. The heterogeneity of the genetic targets and the quality of the studies (which ranged from moderate to high according to the JBI checklist) meant that it was not possible to carry out a meta-analysis.

Investigations on *CD14* rs2569190, *CXCR2*, and *miR-27a-3p* rs895819 polymorphisms suggested possible associations with peri-implantitis risk. However, it should be noted that the findings were primarily derived from single-country case–control studies with limited sample sizes (<200 participants) and lacked replication in independent populations. The differences in the methods of DNA extraction, the platforms used for genotyping, and the adjustment variables further complicated cross-study synthesis. Levels of endothelin-1 (*ET-1*) have been shown to be elevated in cases of peri-implant mucositis and peri-implantitis in comparison with healthy sites, thus supporting its potential as an early biomarker. Nevertheless, a number of methodological constraints preclude its incorporation into a quantitative synthesis.

The subgroup addressing matrix metalloproteinases (*MMP-1*, *MMP-3*, *MMP-7*, *MMP-13*, *TIMP2*) presented both positive and null findings. It was observed that there were population-specific effects and that there was significant heterogeneity with regard to gene–environment interactions, particularly in relation to smoking and history of periodontitis. The presence of these inconsistencies precludes the derivation of a meaningful pooled OR. The association between the *MMP-8* rs11225395 (T allele) and increased susceptibility to peri-implantitis in a Chinese Han cohort is undermined by methodological limitations, which compromise cross-study comparability. The sample size was moderate and restricted to a single ethnic population, which is a limitation in terms of external validity.

### 4.4. Limitations

The evidence presented in this review is subject to several limitations that should be acknowledged.

Firstly, the included studies demonstrated substantial heterogeneity in study design, population selection, and diagnostic criteria for peri-implantitis, which complicated direct comparison and quantitative pooling. The definitions of peri-implantitis and mucositis were found to vary, especially with regard to probing depth, bleeding thresholds, and radiographic bone loss criteria.

Secondly, the sample sizes were generally small, and the majority of studies were conducted in single ethnic populations, which limits the external validity of the research.

Thirdly, a significant number of studies did not control for confounding factors such as smoking status, diabetes, systemic inflammation, or history of periodontitis, which have been demonstrated to influence both genetic expression and disease outcomes. Furthermore, discrepancies in laboratory techniques (e.g., PCR, ELISA, RT-qPCR, genotyping platforms) and the absence of standardized reporting of allele or genotype frequencies resulted in reproducibility and comparability issues.

Finally, the critical appraisal according to the Joanna Briggs Institute (JBI) revealed that several of the included studies exhibited a moderate risk of bias due to inadequate multivariate adjustment, absence of blinding, and population-specific designs.

It is recommended that future research concentrate on larger, multiethnic cohorts, standardized diagnostic criteria, and consistent reporting of genotypic data, including gene–environment interactions. The integration of biochemical biomarkers with genetic polymorphism analysis has the potential to enhance diagnostic accuracy and facilitate a more comprehensive understanding of peri-implant disease susceptibility.

## 5. Conclusions

The increasing research developed on IP highlights the multifactorial nature of this disease, where genetic predisposition seems to play a crucial role, together with environmental and microbial factors. Several polymorphisms in genes involved in inflammatory and immune system pathways have been associated with an increased risk of developing IP, as *IL-1β* +3954 C/T, *CD14* rs2569190, and *TNF-α* −308 G/A, suggesting that the host response contributes to disease progression.

With the results of several studies in the field of molecular genetics in IP, the genetic biomarkers appear to be strongly associated with an increased risk of developing IP; others, such as *IL-1α*, *IL-1β*, *TNF-α*, *MMP8*, *OPG*, and inflammatory cytokines, demonstrate some variability or inconsistent results in modulating immune responses and consequently influencing peri-implant bone loss.

The inclusion of the *miR-27a-3p* rs895819 polymorphism as an additional risk factor reinforces the need for personalized approaches in diagnosis and treatment, considering genetic predispositions alongside environmental and systemic factors. Endothelin-1 (*ET-1*) has been identified as a potential early biomarker in peri-implant diseases, including mucositis and peri-implantitis, indicating the presence of early inflammatory activity. Future research should aim to integrate genetic data with advanced biomarker technologies to refine diagnostic strategies and optimize therapeutic interventions for peri-implantitis.

Numerous studies, which are in turn more specific, on genetic polymorphisms and the different expressions of cytokines in the field of IP, in conjunction with the inclusion of new medicines, suggest the existence of promising avenues for the prevention of the disease and targeted therapies.

New research on genetic association and peri-implantitis with larger sample sizes and population diversity, better statistical control of confounders, multivariate analyses to confirm the association, and standardized procedures to make comparability easier are all advised, considering the study’s findings.

This emerging evidence highlights the importance of genetic screening integrated with clinical evaluations in order to guide personalized treatment strategies. Future studies should focus on large-scale and advanced molecular techniques to refine the understanding of genetic contributions to IP and enhance targeted therapeutic intervention.

## Figures and Tables

**Table 1:** PICO Strategy.

Patient	Individuals receiving dental implants
Intervention	Dental implant placement
Comparison	Genetic alterations vs. absence associated with peri-implantitis
Outcome	Susceptibility to developing peri-implantitis

**Table 2:** Summary of findings on genetic polymorphisms and susceptibility to peri-implantitis.

Author/Year	Study Type	Population (Country)	Gene/Polymorphism	Main Finding	Effect
Gonçalves Junior et al., 2016 [7]	Prospective	Brazil	*MMP13*, *TIMP2*, *TGFB3*	No significant associations found	No association
He K. et al., 2020 [6]	Case–control	China	*IL-1A* (−889 C/T), *IL-1B* (+3954 C/T), *TNF-α* (−308 G/A)	*IL-1A* and *IL-1B* polymorphisms increase risk; *TNF-α* not significant	Increased risk
Zhou et al., 2016 [4]	Case–control	China	*OPG* rs2073617, rs2073618	*CC* genotype (rs2073618) linked to higher IP risk; rs2073617 not significant	Increased risk
Ribeiro et al., 2017 [1]	Case–control	Brazil	*IL-10* (−1082 A/G), *RANKL* (−438 A/G)	No association with implant failure	No association
Silva et al., 2020 [13]	Case–control	Brazil	*RANK*, *RANKL*, *OPG*	No significant associations; environmental factors more influential	No association
Rakic et al., 2015 [9]	Population-based	Southeastern Europe	*CD14*-159 C/T, *TNFα* (−308 A/G)	CC *(CD14*) and AG (*TNFα*) genotypes associated with 5-fold higher risk of IP	Increased risk
Coelho et al., 2016 [14]	Prospective	Brazil	*BMP4*, *FGF3*, *FGF10, FGFR1*	*BMP4* and *FGF10* haplotypes associated with IP; *FGF3* TT + CT genotypes linked to healthy implants	Increased risk
Chang et al., 2021 [11]	Case–control	China	*EGF* rs2237051, rs4444903	*GG* genotype (rs2237051) protective; (rs4444903) not significant	Protective effect
Cardoso et al., 2022 [15]	Case–control	Portugal	*IL-1A* (−889), *IL-1B* (+3954)	Mutated alleles more frequent in IP group	Increased risk
Petkovic-Curcin et al., 2017 [8]	Case–control	Serbia	*CD14*, *TNFα*, *IL6*, *IL10, IL1ra*	*TNFα*-308 GA/AA increase risk; *CD14*-159 CT/TT protective	Mixed (Risk and Protective)
Saremi et al., 2021 [16]	Case–control	Iran	*IL-10* (−819 C/T, −592 C/A), *IL-1ß* (+3954 C/T), *TNF-α* (−857 G/A, −308 G/A)	*IL-10* and *IL-1ß* variants increase risk; *TNF-α* not associated	Increased risk
Turkmen et al., 2022 [3]	Prospective	Turkey	*FPR1*	G/C genotype associated with higher IP risk	Increased risk
Li et al., 2024 [10]	Case–control	China	*CD14* rs2569190	GG genotype and G allele increase risk	Increased risk
Talib et al., 2024 [17]	Cross sectional	Iraq	*IL-17A* rs2275913, *IL-23*	A/A and G/A genotypes increase risk; *IL-23* elevated in IP	Increased risk
Giro et al., 2023 [2]	Case–control	Brazil	*IL-4*, *MIP-1α*, *MMP-9*	*IL-4* expression 18× higher in IP tissues	Increased risk
Gao et al., 2025 [18]	Case–control	China	*miR-27a-3p* rs895819	GG genotype and G allele increase IP susceptibility	Increased risk
Qi et al., 2021 [19]	Prospective	China	*CXCR2* rs2230054, rs1126580	CT and AG genotypes increase risk of IP	Increased risk
Saremi et al., 2024 [20]	Case–control	Iran	*MMP-1*, *-2*, *-3*, *-7*, *-13*	*MMP-3* and *MMP-7* variants increase risk	Increased risk
Martins et al., 2022 [21]	Case–control	Brazil	*AhR*, *IL-22*, *IL-6*	Higher *AhR* and *IL-6* expression in IP tissues	Increased risk
Jin et al., 2025 [22]	Case–control	China	*MMP-8* rs11225395	T allele (TC/TT) increases risk of IP susceptibility; higher *MMP-8* expression	Increased risk
Fragkioudakis et al., 2025 [23]	Cross sectional	Greece	*MMP-8* (799C/T, −381A/G, +17C/G)	T allele (−799C/T) associated with IP	Increased risk
Lafuente Ibañez de Mendoza et al., 2024 [12]	Case–control	Spain	*GBP1* rs7911, *BRINP3* rs1935881, *OPG* rs2073617, *BMP-4* rs17563, *FGF-3* rs1893047	*SNPs* linked to IP, especially in smokers and diabetics	Increased risk
Saito et al., 2024 [24]	Cross sectional	Japan	*ET-1*, *IL-1β*	*ET-1* elevated in IP/mucositis; potential diagnostic biomarker	Increased risk

**Table 3:** Data extracted from the selected studies.

Author/Year	Type of Study	Objective	Population	Results	Conclusions
Rakic et al., 2015 [9]	Population Study	To investigate whether the *SNPs CD14*-159 C/T and *TNFα* −308 A/G are associated with IP.	Southeastern European Caucasians	Analysis of the *CD14*-159 C/T polymorphism showed that the CC genotype was associated with IP, with a five-fold increased risk in these carriers. For the *TNFα*−308 A/G polymorphism, the AG genotype was also associated with IP, with a five-fold increased risk.	The *CD14*-159 C/T and *TNFα* −308 A/G polymorphisms are associated with IP and may represent biomarkers for peri-implant disease.
Coelho et al., 2016 [14]	Prospective Study	Analyzing the correlation between the *BMP4*, *FGF3*, *FGF10* and *FGFR1* genes and peri-implant bone loss.	Brazilian population	The TT polymorphic genotype for *BMP4* rs2761884 was associated with healthy peri-implants. The *FGF3* rs4631909 genotype (TT + CT genotypes) also showed an association with the control group. The frequency of the C allele for *FGF3 rs4631909* showed a trend towards association with IP. The haplotypes *FGF10* CCTG (*p* = 0.03), *BMP4* GAAA (*p* = 0.05), and GGGA (*p* = 0.02) were associated with IP.	The BMP4 and FGF10 haplotypes are associated with IP.
Gonçalves Junior et al., 2016 [7]	Prospective Study	To evaluate possible associations between polymorphisms in the *MMP13*, *TIMP2*, and *TGFB3* genes and IP.	Brazilian population	No significant associations were found between the polymorphisms in the genes studied and the development of IP.	The polymorphisms studied showed no direct relationship with IP.
Zhou et al., 2016 [4]	Case–control Study	To evaluate the association between the *T950C* (rs2073617) and *G1181C* (rs2073618) polymorphisms of the *OPG* gene and susceptibility to IP	Chinese Han population	The results of the study showed that people with the CC genotype of rs2073618 are more likely to have IP than carriers of the GG genotype. In addition, patients with the C allele have a 1.47 times greater risk of developing IP, but there was no association with the rs2073617 polymorphism. The frequency of the G-C haplotype of rs2073618-rs2073617 was significantly correlated with greater susceptibility to IP.	The *OPG* rs2073618 polymorphism may be related to a risk of IP, but not rs2073617.
Petkovic-Curcin et al., 2017 [8]	Case–control study	Analyze the association of polymorphisms in cytokine genes (*CD14, TNFα*, *IL6*, *IL10, IL1ra*) with the risk of IP.	Serbian population	Smokers (OR 3.28) and history of periodontitis (OR 6.33) increased the risk of IP; *TNFα*-308 GA/AA associated with higher risk (OR 8.89) and *CD14*-159 CT/TT protective genotype (OR 0.059).	Smoking and the *TNFα*-308 GA/AA genotype increase risk, while *CD14*-159 CT/TT reduces susceptibility to peri-implantitis.
Ribeiro R. et al., 2017 [1]	Case–control Study	Investigate the association between *IL-10* (−1082 A/G) and *RANKL* (−438 A/G) genetic polymorphisms and dental implant failure.	Brazilian population	No statistically significant difference was found between implant failure and the genotypes or allele frequencies of *IL-10* (−1082) or *RANKL* (−438).	No association was found between the *RANKL* (−438) and *IL-10* (−1082) genetic polymorphisms and implant failure.
He K. et al., 2020 [6]	Case–control Study	To ascertain the association between genetic polymorphisms in the *TNF-α* (−308 G/A), *IL-1A* (−889 C/T), and *IL-1B* (+3954 C/T) genes and the risk and severity of peri-implantitis in a non-smoking population.	Chinese population	Genotypes with the *T* allele in *IL-1A* (−889 C/T) and *IL-1B* (+3954 C/T) are significantly associated with an increased risk of peri-implantitis (ORs: 2.0–2.5 for CT and TT vs. CC). The *TNF-α* (−308 G/A) polymorphism showed no significant association.	The IL-1A −889C/T and IL-1B +3954C/T polymorphisms appear to be genetic markers of susceptibility to peri-implantitis, also influencing clinical severity; meanwhile, the TNF-α −308G/A polymorphism does not appear to be relevant.
Silva et al., 2020 [13]	Case–control Study	Investigate polymorphisms in *RANK* (rs3826620), *RANKL* (rs9594738), and *OPG* (rs2073618) and their relationship with mucositis and peri-implantitis.	Brazilian population	There was no significant association between polymorphisms and peri-implantitis/mucositis.	In this population, clinical and environmental factors have a greater influence than genetic polymorphisms on the development of peri-implantitis.
Qi et al., 2021 [19]	Prospective study	To investigate the role of chemokine receptor 2 *(CXCR2*) gene polymorphisms in IP susceptibility.	Chinese Han population	The CT genotype of rs2230054 and the AG genotype and G allele of rs1126580 significantly increased in IP patients compared with healthy implants.	The *CXCR2* gene rs220054 and rs1126580 polymorphisms were associated with the IP susceptibility. The CT genotype of rs2230054 and the AG genotype and G allele of rs1126580 serve as risk factors for the occurrence of IP.
Saremi et al., 2021 [16]	Case–control Study	Evaluate the frequency of *SNPs* in the *IL-10*, *IL-1ß*, and *TNF-α* genes in patients with IP and healthy controls.	Iranian population	The analysis revealed that the allele and genotype frequencies of *IL-10* −819 C/T, *IL-10* −592 C/A, and *IL-1ß* + 3954 C/T differed significantly between patients with IP and healthy controls. However, no significant association was observed between the *TNF-α* −857 G/A and *TNF-α* −308 G/A polymorphisms and IP.	The genetic polymorphisms *IL-10* −819 C/T, *IL-10* −592 C/A, and *IL-1ß* + 3954 C/T may play a role in the pathogenesis of IP and increase the risk of its occurrence.
Chang et al., 2021 [11]	Case–control Study	The association of epidermal growth factor (*EGF*) gene polymorphisms with susceptibility to IP.	Chinese population	The GG genotype and the G allele of rs2237051 proved to be more frequent in the IP group compared to the healthy implant group. Compared to carriers of the AA genotype, carriers of the GG genotype of rs2237051 had a lower risk of IP. There was no significant difference in the rs4444903 genotype between cases and controls.	The *EGF* rs2237051 polymorphism showed a significant association with IP. The GG genotype of rs2237051 and the G allele may be protective factors for the development of IP.
Martins et al., 2022 [21]	Case–control Study	It evaluated the expression of *AhR*, *IL-22*, and *IL-6* in the peri-implant tissues of healthy patients and those with IP.	Brazilian population	Higher levels of *AhR* and *IL-6* expression were observed in the IP tissues. *IL-22* expression levels did not differ between the groups.	Higher levels of *AhR* and *IL-6* expression were detected in the soft tissues of patients with IP.
Turkmen et al., 2022 [3]	Prospective study	To assess whether there is a solid genetic predisposition that causes the formation of IP.	Turkish population	The polymorphism of the *fMLP* receptor gene (*FPR1*) creates a significant difference in individuals with a higher risk of IP.	The results showed that individuals with the G/C genotype have a higher risk of IP.
Cardoso et al., 2022 [15]	Case–control Study	To assess whether individuals with *SNPs* in the *IL-1A* (rs1800587) and *IL-1B* (rs1143634) genes are more susceptible to developing IP.	Portuguese population	For the *IL-1A* polymorphism (−889), it was observed that the mutated allele was present in a higher percentage in the IP group compared to the control group. For the *IL-1B* polymorphism (+3954), the altered allele was also present in a higher percentage in the IP group than in the control group.	There is an association between the *IL-1A* (−889) and *IL-1B* (+3954) polymorphisms and IP.
Giro et al., 2023 [2]	Case–control Study	Evaluate the expression levels of *IL-4*, *MIP-1α*, and *MMP-9* in healthy peri-implant tissue and in IP.	Brazilian Population	*IL-4* expression showed higher values (18×) in the group of patients with IP compared to the healthy group.	In the tissues affected by IP, only *IL-4* levels were increased when compared to the tissues in the control group.
Li et al., 2024 [10]	Case–control Study	To assess the genetic correlation of *CD14* gene polymorphisms with predisposition to IP.	Chinese population	A high percentage of carriers of the *GG* rs2569190 genotype or *G* allele was observed in the IP group compared to the control group. Carriers of the *GG* rs2569190 genotype had a higher risk of developing IP.	The rs2569190 polymorphism of the *CD14* gene was associated with a predisposition to IP in the Chinese population, with the GG genotype and the G allele being risk factors for the development of IP.
Lafuente Ibañez de Mendoza et al., 2024 [12]	Case–control Study	To evaluate whether certain single-nucleotide polymorphisms *(SNPs*) of genes related to inflammation and bone metabolism are associated with the presence of peri-implantitis.	Spanish population	The *GBP1* rs7911 and *BRINP3* rs1935881 SNPs were significantly more frequent in patients with IP. In patients with IP who smoked >10 cigarettes/day, a higher prevalence of the *OPG* rs2073617 SNP was observed. In patients with IP and type II diabetes mellitus, the *BMP-4* rs17563 and *FGF-3* rs1893047 SNPs were more frequent.	*SNPs* in *GBP1, BRINP3, OPG, BMP-4*, and *FGF*-3 could serve as risk markers, especially in subgroups with comorbidities (diabetes) or habits (smoking).
Saremi et al., 2024 [20]	Case–control Study	Evaluating the association between genetic polymorphisms of matrix metalloproteinases *(MMP-) 1*, *-2*, *-3*, *-7*, and *-13* with IP.	Iranian population	The *MMP-3* (−1171 5A/6A) and *MMP-7* (−181 A/G) polymorphisms showed significant differences between patients with IP and healthy controls. However, the genetic polymorphisms of *MMP-1* (−1607 1G/2G), *MMP-2* (−1306 C/T), and *MMP-13* (−77 A/G) showed no differences in prevalence between the two groups.	The *MMP-3* (−1171 5A/6A) and *MMP-7* (−181 A/G) polymorphisms showed differences when comparing patients with IP and healthy controls from the study population.
Talib et al., 2024 [17]	Cross-sectional study	To identify the role of the immune-genetic variation in *IL*-*17A* and related inflammatory cytokine (*IL-23*) in the initiation and progression of IP.	Iraqi population	A significant elevation in the mean level of *IL-23* in the IP patient group, higher than its level in the successful implant and control groups, was observed. The A/A and G/A genotypes were significantly associated with IP increased risk.	*IL-17A* gene polymorphism may play a role in IP disease susceptibility, especially in persons carrying the rs2275913 A allele at a higher risk of developing IP as compared with those carrying the G allele.
Saito et al., 2024 [24]	Cross-sectional Study	To evaluate the potential of Endothelin-1 (*ET-1*) as a biomarker for diagnosing peri-implant diseases.	Japanese population	*ET-1* levels were significantly elevated in the IP compared with the healthy group, and highest in the IP mucositis group. *IL-1β* levels were significantly higher in the IP group than in the healthy group. *ET-1* exhibited superior area under the curve values, sensitivity, and specificity compared to those of *IL-1β.*	The presence of *ET-1* plays a role in IP diseases. Its significantly increased expression IP mucositis indicates its potential for enabling earlier and more accurate assessments of IP inflammation when combined with conventional examination methods.
Fragkioudakis et al., 2025 [23]	Cross-sectional study	To explore the relationship between matrix metalloproteinase−8 (*MMP*−*8*) gene polymorphisms (−799C/T, −381A/G, and +17C/G) and IP.	Greek population	The −799C/T polymorphism significantly associated with IP, with T allele carriers having a higher diagnosis rate. Although T allele carriers exhibited higher mean values for the probing depth, clinical attachment level, and bleeding on probing, these differences were not statistically significant across genotypes. No associations found between the −381A/G and +17C/G polymorphisms and IP clinical parameters.	The −799C/T polymorphism, specifically the T allele, is strongly linked to IP, indicating its potential as a genetic marker for disease susceptibility.
Jin et al., 2025 [22]	Case–control study	To investigate the association between the *MMP-8* rs11225395 polymorphism and IP in the Chinese Han population.	Chinese Han population	Rs11225395 T allele was significantly associated with an increased risk of IP, with the TC/TT genotype exhibiting higher susceptibility. *MMP-8* expressions were upregulated in IP patients. The rs11225395 locus, individuals with the TC/TT genotype showed a significantly higher relative expression level of *MMP-8.*	The *MMP-8* rs11225395 polymorphism was significantly associated with genetic susceptibility to IP.
Gao et al., 2025 [18]	Case–control study	To investigate the relationship between *miR-27a-3p* rs895819 polymorphism with IP susceptibility.	Chinese population	GG genotype and G allele of rs895819 demonstrate a significant association with an enhanced IP susceptibility.	The *rs895819* of *miR-27a-3p* serves as a significant risk predictor for IP patients.

**Table 4:** Risk of bias assessment by Joanna Briggs Institute checklist.

Author and Year	1	2	3	4	5	6	7	8	9	10	Score	Quality
Rakic et al., 2015 [9]	Y	Y	N	Y	Y	?	?	Y	N/A	Y	6	Moderate
Coelho et al., 2016 [14]	Y	Y	Y	Y	Y	?	?	Y	N/A	Y	7	High
Gonçalves Junior et al., 2016 [7]	Y	Y	N	Y	Y	?	?	Y	N/A	Y	6	Moderate
Zhou et al., 2016 [4]	Y	Y	Y	?	Y	?	?	Y	?	Y	6	Moderate
Petkovic-Curcin et al., 2017 [8]	Y	Y	Y	Y	Y	Y	Y	Y	Y	Y	10	High
Ribeiro R et al., 2017 [1]	Y	P	?	Y	Y	?	P	?	Y	P	5.5	Moderate
He K. et al., 2020 [6]	Y	Y	Y	Y	Y	N	?	Y	?	Y	7	High
Silva et al., 2020 [13]	Y	Y	Y	Y	Y	P	Y	Y	Y	Y	9.5	High
Chang et al., 2021 [11]	Y	Y	N	Y	Y	?	?	Y	N/A	Y	6	Moderate
Qui et al., 2021 [19]	Y	Y	N	Y	Y	?	?	Y	N/A	Y	6	Moderate
Saremi et al., 2021 [16]	Y	Y	Y	Y	Y	?	?	Y	N/A	Y	7	High
Martins et al., 2022 [20,21]	Y	Y	Y	Y	Y	?	?	Y	N/A	Y	7	High
Turkmen et al., 2022 [3]	Y	Y	N	Y	Y	?	?	Y	N/A	Y	6	Moderate
Cardoso et. al., 2022 [15]	Y	Y	Y	Y	Y	?	?	Y	N/A	Y	7	High
Giro et al., 2023 [2]	Y	Y	Y	Y	Y	?	?	Y	N/A	Y	7	High
Li et al., 2024 [10]	Y	Y	N	Y	Y	?	Y	Y	N/A	Y	7	High
Saremi et al., 2024 [20]	Y	Y	Y	Y	Y	?	?	Y	N/A	Y	7	High
Talib et al., 2024 [17]	Y	Y	Y	Y	Y	?	?	Y	N/A	Y	7	High
Lafuente Ibañez et al., 2024 [12]	Y	Y	?	Y	Y	Y	Y	?	Y	Y	8	High
Saito I. et al., 2024 [24]	Y	Y	Y	P	Y	N	N	Y	?	Y	6.5	Moderate
Jin H. et al., 2025 [22]	Y	Y	Y	Y	Y	Y	Y	N	?	Y	8.5	High
Fragkioudakis I. et al., 2025 [23]	Y	Y	?	Y	Y	Y	?	N	Y	Y	8	High
Gao et al., 2025 [18]	Y	Y	Y	Y	Y	?	?	Y	N/A	Y	7	High

Note: The evaluation score ranges: 1 (lowest) to 10 (highest); Y = Yes; N = No; P = Partial; ? = Uncertain; N/A = Not applicable. The quality groups: Low: 0–3, Moderate: 4–6, and High: 7–10. Of the 23 included studies, 8 were classified as moderate quality (35%) and 15 were classified as high quality (65%). The results indicate that the articles included exhibit a high level of methodological compliance and a solid basis for trusting the overall results, with a certain risk of bias and variability in quality among the articles.

## Data Availability

No new data were created or analyzed in this study. Data sharing is not applicable to this article.

## Abbreviations

The following abbreviations are used in this manuscript:

AhR	Aryl Hydrocarbon Receptor
BMP	Bone Morphogenetic Protein
BRIMP	Bone Morphogenetic/Retinoic Acid-Inducible Neural-Specific Protein
CD	Differentiation Cluster
CRP	C-reactive Protein
CXCR	C-X-C Chemokine Receptor
DNase I	Deoxyribonuclease I
EGF	Epidermal Growth Factor
ELISA	Enzyme-linked Immunosorbent Assay
ET-1	Endothelin 1
FGF	Fibroblast Growth Factor
FGFR	Fibroblast Growth Factor Receptor
fMLP	N-Formyl-Methionyl-Leucyl-Phenylalanine
FPR	Formyl Peptide Receptor
GBP	Guanylate-Binding Proteins
GPCR	G protein-coupled Receptor
GWAS	Genome-wide Association Study
IL	Interleukin
IP	Peri-implantitis
JBI	Joanna Briggs Institute
MMP	Matrix Metalloproteinase
ORs	Odds Ratios
OPG	Osteoprotegerin
PCR	C-reactive Protein Test
PICO	Patient, Intervention, Comparison, Outcome
RANK	Receptor Activator of Nuclear Factor xB
RANKL	Receptor Activator of Nuclear Factor xB Ligand
RT-qPCR	Real-time Reverse Transcription Reaction
RUNX	Runt-related Transcription Factor
SNP	Single-Nucleotide Polymorphism
TGF	Tumor Growth Factor
TIMP	Tissue Inhibitor of Metalloproteinases
TNF	Tumor Necrosis Factor
WBC	White Blood Cell
